# A review of the association between baseline [^18^F] fluorodeoxyglucose uptake and axillary pathological complete response in node-positive breast cancer patients: focus on clinical subtypes

**DOI:** 10.1097/MNM.0000000000001964

**Published:** 2025-02-24

**Authors:** Melissa Lenaerts, Florien J. G. van Amstel, Felix M. Mottaghy, Sandra M. E. Geurts, Vivianne C. G. Tjan-Heijnen, Marjolein L. Smidt, Thiemo J. A. van Nijnatten

**Affiliations:** a Department of Surgery, Maastricht University Medical Centre+,; b GROW – Research Institute for Oncology and Reproduction, Maastricht University,; c Department of Radiology and Nuclear Medicine, Maastricht University Medical Centre+, Maastricht, The Netherlands,; d Department of Nuclear Medicine, University Hospital RWTH Aachen University, Aachen, Germany and; e Department of Medical Oncology, Maastricht University Medical Centre+, Maastricht, The Netherlands

**Keywords:** [^18^F] fluorodeoxyglucose PET/computed tomography, axillary lymph nodes, axillary pathologic complete response, breast cancer, clinical subtypes, metabolic activity, neoadjuvant systemic therapy

## Abstract

The objective is to assess whether the degree of metabolic uptake of the primary tumor and axillary lymph nodes (ALNs) on baseline [^18^F] fluorodeoxyglucose ([^18^F]FDG) PET/CT is associated with the probability to achieve axillary pathologic complete response (pCR) in clinically node-positive (cN+) breast cancer patients treated with neoadjuvant systemic therapy (NST), overall and per clinical subtype. Studies that assessed the maximum standardized uptake value (SUVmax) in the primary tumor and ALNs on baseline [^18^F]FDG PET/CT and reported axillary pCR rates in patients diagnosed with cN+ invasive breast cancer treated with NST, followed by surgery, were searched. Area under the curve (AUC) values were obtained. A total of seven studies (561 patients) were included. The mean baseline SUVmax of the primary tumor ranged from 8.1 (±4.3) to 9.8 (±7.2). Mean baseline axillary SUVmax ranged from 6.0 (±5.6) to 7.3 (±6.2). The axillary pCR rate ranged from 38.0% to 48.1%. Considering the primary tumor, no study reported on the association between baseline SUVmax and the axillary pCR rate. Considering the ALNs, the AUC value for baseline axillary SUVmax to predict axillary pCR ranged from 0.52 [95% confidence interval (CI): 0.39–0.65; all subtypes included] to 0.74 (95% CI: 0.53–0.95; only human epidermal growth factor receptor 2+ and triple negative). In conclusion, no association between the primary tumor SUVmax on baseline [^18^F]FDG PET/CT and axillary pCR was found. Concerning the axilla, based on limited scientific evidence, the axillary SUVmax on baseline [^18^F]FDG PET/CT may be associated with axillary pCR after NST in cN+ breast cancer patients, however, potential differences between clinical subtypes should be considered.

## Introduction

In recent years, systemic therapy in early-stage breast cancer has increasingly shifted from the adjuvant to the neoadjuvant setting [i.e. neoadjuvant systemic therapy (NST)] [1]. This shift allows for response monitoring and enables less invasive surgery in case of response to NST in the breast and/or axillary lymph nodes (ALNs) [2]. Axillary pathologic complete response (pCR) after NST in clinically node-positive (cN+) patients is observed in 13–60%, depending on the clinical subtype of breast cancer [3].

Imaging by using PET/computed tomography (PET/CT) with [^18^F] fluorodeoxyglucose ([^18^F]FDG) as PET tracer is often considered at the time of diagnosis to exclude distant metastasis in cN+ patients [4,5]. [^18^F]FDG PET/CT exams provide information on the metabolic activity of cancer cells by measuring standardized uptake values (SUVs) [4]. There is a substantial variability in baseline SUVs in both the primary tumor and ALNs. For the primary tumor, no difference in baseline SUVs between patients with and without breast pCR was reported so far [6,7]. Nevertheless, a recent meta-analysis demonstrated that the degree of metabolic uptake within the primary breast tumor differs among clinical subtypes, with significantly lower baseline SUVs in the estrogen receptor (ER)+ subtype compared with human epidermal growth factor receptor 2 (HER2)+ and triple negative (TN) subtypes [8]. For the ALNs, it is known that a hypermetabolic ALN on baseline [^18^F]FDG PET/CT can be considered malignant, but it remains unclear whether the degree of metabolic uptake is associated with the probability to achieve axillary pCR to NST and whether it differs among clinical subtypes.

Therefore, this systematic review aims to assess the degree of metabolic uptake in the primary tumor and ALNs on baseline [^18^F]FDG PET/CT in association with axillary pathologic response in cN+ breast cancer patients treated with NST, with a specific focus on clinical subtypes.

## Methods

### Literature search strategy

This systematic review was conducted in accordance with the Preferred Reporting Items for Systematic Review and Meta-Analysis (PRISMA) guidelines. PubMed and Embase were searched up to 31 July 2024 for eligible studies concerning the association between the degree of metabolic uptake in primary tumor and ALNs on [^18^F]FDG PET/CT and axillary pCR. Details concerning the search strategy can be found in Supplementary Tables S1 and S2, Supplemental digital content 1, http://links.lww.com/NMC/A325. Two researchers (M.L. and F.J.G.A.) independently screened all studies by title and abstract. Next, the full text of the remaining studies was read and checked for eligibility. A consensus meeting was held to discuss discrepancies. For the nonresolved discrepancies, a third reviewer (T.J.A.N.) was consulted for a final call.

### Study inclusion criteria

Studies were eligible if they included patients diagnosed with cN+ invasive breast cancer (evaluated by pathology or [^18^F]FDG PET/CT), treated with NST (chemo- with or without targeted therapy), who underwent [^18^F]FDG PET/CT at baseline, for which the maximum standardized uptake value (SUVmax) of the primary tumor and ALNs was assessed. After NST, patients underwent axillary surgical staging (sentinel lymph node biopsy, marking axillary lymph node with radioactive iodine seed, or targeted axillary dissection) and/or axillary lymph node dissection to determine axillary pCR. Eligible studies had to be written in English and had to be available in full text. Studies were excluded in the cases of inflammatory breast cancer, clinically node-negative status (cN0), or distant metastases. Furthermore, all reviews, meta-analyses, editorials, case reports, technical reports, letters, and studies with duplicated results were excluded.

### Outcome measures

First, the degree of metabolic uptake for the primary tumor and ALNs overall and for clinical subtypes specifically on baseline [^18^F]FDG PET/CT was reported. Second, axillary pCR rates overall and for clinical subtypes were assessed. Third, the degree of metabolic uptake in the primary tumor and ALNs overall and for clinical subtypes on baseline [^18^F]FDG PET/CT was associated with axillary pCR.

### Data extraction and quality assessment

Two reviewers (M.L. and F.J.G.A.) extracted the following study characteristics from eligible articles independently: first author, year of publication, study design, included clinical subtypes, sample size, method of cN+ diagnosis (by pathological or baseline [^18^F]FDG PET/CT confirmation), type of PET/CT scanner including PET/CT exam settings, type of NST, and type of axillary surgery procedure. Included subtypes comprise ER+/HER2−, HER2+, TN, HER2+/TN (grouped), Luminal A, and Luminal B. Patients with HER2+ tumors were allowed to receive additional targeted therapy (trastuzumab or a combination of trastuzumab and pertuzumab). Maximum blood glucose concentrations expressed in mg/dl were recalculated into mmol/L using the following formula: $mmol/L\;=\;\frac{mg/dl}{18}$. In the case of dual time point [^18^F]FDG PET/CT exams, the first exam (i.e. 50–60 min after [^18^F]FDG injection) was extracted. Furthermore, [^18^F]FDG PET/CT-specific characteristics were extracted: baseline SUVmax of the primary tumor and ALNs, overall and differentiated per clinical subtype. SUVmax expressed as median (range) were recalculated into mean ± SD [9,10]. In case a study population consisted of cN0 and cN+ patients, only the SUVmax of the cN+ patients were extracted. In case the differentiation between cN0 and cN+ was not feasible, the study was excluded from this systematic review. Moreover, the percentage of patients that achieved axillary pCR post-NST was extracted and the diagnostic performance parameters such as sensitivity, specificity, positive predictive value (PPV), negative predictive value (NPV), and corresponding area under the curve (AUC) values were obtained when available. The quality of all included studies was assessed individually by two researchers (M.L. and F.J.G.A.) using the Quality Assessment of Diagnostic Accuracy Studies 2 (QUADAS-2) tool.

## Results

### Study selection

**Fig. 1:**
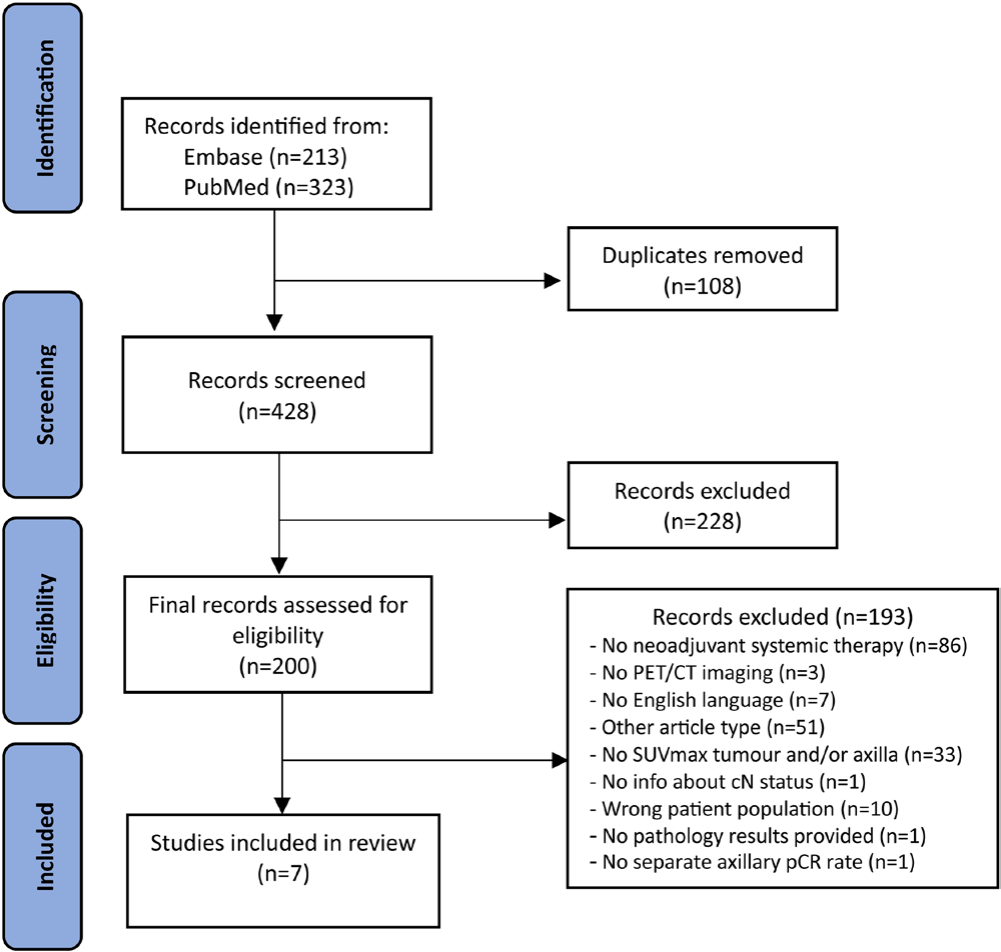
Flow diagram illustrating the study selection process (PRISMA). PRISMA, Preferred Reporting Items for Systematic Review and Meta-Analysis.

The literature searches on PubMed and Embase identified 536 publications. After removing 108 duplicates, 428 abstracts were screened that resulted in the selection of 200 papers for full text review. Of those, seven studies met the inclusion criteria and were included in this review (Fig. 1).

### Quality of included studies

**Fig. 2:**
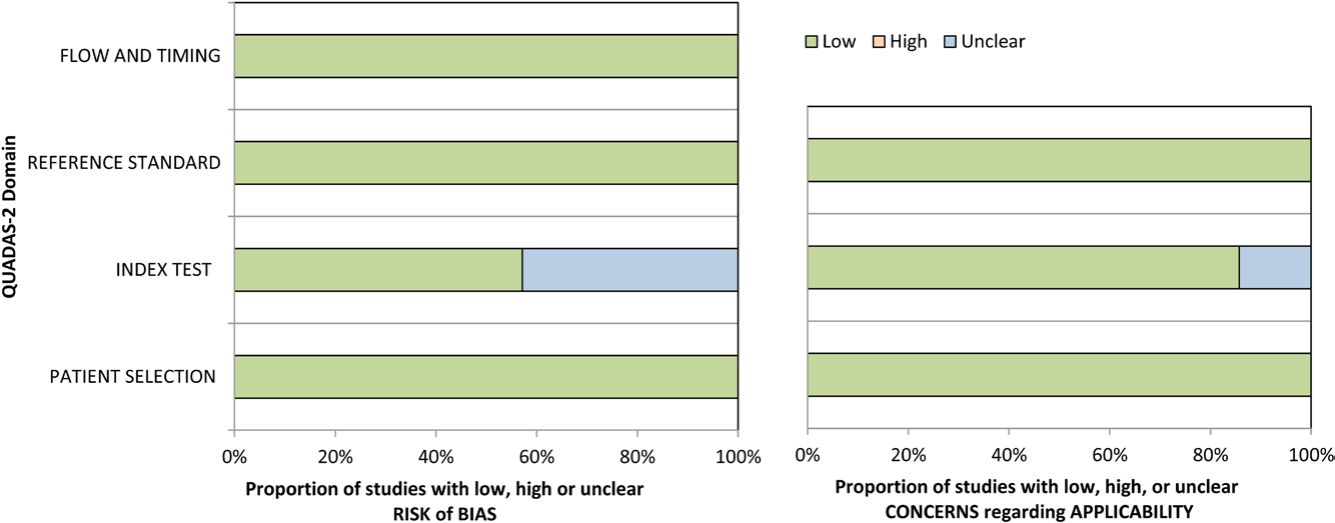
QUADAS-2 tool. Evaluation of risk of bias and applicability of primary diagnostic studies.

Figure 2 provides an overview of the risk of bias and concerns regarding the applicability of included studies. Three studies had an unclear risk of bias for the index test. For applicability, no high concerns were reported.

### General study characteristics

The seven studies included a total of 561 female patients (range: 22–180) treated between 2008 and 2021. In five studies (495 patients), initial cN+ disease before NST was histopathologically confirmed [11–15]. In the remaining two studies (66 patients), initial cN+ disease before NST was based on baseline [^18^F]FDG PET/CT exams [16,17]. The [^18^F]FDG PET/CT exam settings, regimen of NST, and axillary surgery procedure varied within the studies (Table 1).

**Table 1:** Overview of study characteristics

First author	Year	Study design	Sample size	Included clinical subtypes	cN diagnosis	[^18^F]FDG PET/CT scanner	[^18^F]FDG PET/CT exam settings					NST regimen	Axillary surgery procedure
							Use of EARL ^18^FDG PET/CT reconstruction	Tracer dose (MBq/kg)	No. bed positions per minute	Max. glucose concentration (mmol/L)	Minutes between FDG injection and PET/CT exam		
Can	2021	Retrospective	180	Luminal A (22/180) Luminal B (111/180) HER2+ (24/180) TN (23/180)	PA	Discovery IQ Siemens biograph 6	No	3.5–5.5		7.8	60	Anthracycline and/or taxane with trastuzumab if HER2+	SLNB ALND
Garcia Vicente	2014	Prospective	76	Luminal A (3/76) Luminal B HER2− (17/76) Luminal B HER2+ (26/76) ER− PR−/HER2+ (11/76) TN (19/76)	PA	Discovery DSTE-16 s	No		3	8.9	60	Anthracycline and/or taxane with trastuzumab if HER2+	ALND (100%)
Groheux	2013	Prospective	22	HER2+ (22/22)	[^18^F]FDG PET/CT	Gemini XL	No	5	2	7	60	Anthracycline and taxane and trastuzumab	ALND (100%)
Koolen	2013	Prospective	80	HER2+ (24/80) ER+/HER2− (34/80) TN (22/80)	PA	Gemini TF	No		3		60 ± 10	Anthracycline and/or platinum with trastuzumab if HER2+	ALND (100%)
de Mooij	2021	Retrospective	52	ER+/HER2− (25/52) HER2+ (12/52) TN (15/52)	PA	Gemini TF	Yes	2	2	10	45–60	Anthracycline, taxane and/or platinum with trastuzumab and/or pertuzumab if HER2+	SLNB TAD ALND
van Ramshorst	2017	Prospective	105	ER+ PR+/HER2+ (31/105) ER− PR−/HER2+ (29/105) TN (45/105)	PA	Gemini TF	Yes		3	10	60 ± 10	Anthracycline, taxane and/or platinum with trastuzumab if HER2+	-ALND (84.7%) -MARI (12.4%)
Tatar	2022	Retrospective	44	Luminal A Luminal B HER2+ TN	[^18^F]FDG PET/CT	Discovery St	No	3.7	7–9	8.3		Anthracycline and/or taxane with trastuzumab and/or pertuzumab if HER2+	SLNB ALND

ALND, axillary lymph node dissection; MARI, marking axillary lymph nodes with radioactive iodine seeds; PA, pathologically; SLNB, sentinel lymph node biopsy; TAD, targeted axillary dissection

— Unknown.

### Baseline maximum standardized uptake value of the primary tumor and axillary lymph nodes

The mean baseline SUVmax (±SD) of the primary tumor ranged from 8.1 (±4.3) to 9.8 (±7.2) [11,17] (Table 2). Differentiated per clinical subtype, the mean baseline SUVmax (±SD) of the primary tumor in the HER2+ subtype ranged from 6.9 (±3.5) to 8 (±4.5), and in the TN subtype, a value of 11.3 (±7.7) was observed [15,16]. No studies reported the mean baseline primary tumor SUVmax in the ER+/HER2− subtype.

**Table 2:** Maximum standardized uptake values of primary tumor and axillary lymph nodes with corresponding axillary pathologic complete response rates

Author	Data type	Baseline SUVmax primary tumor	Baseline SUVmax ALNs	Baseline SUVmax axilla recalculated to mean ± SD	Axillary pCR (%)
Can	Mean ± SD	9.8 ± 7.2	7.3 ± 6.2	7.3 ± 6.2	42.2% (76/180)
Garcia	Mean ± SD		6 ± 5.6	6 ± 5.6	44.7% (34/76)
Koolen	Median (range)		6.1 (1.8–23)	6.9 ± 4.4	38% (30/80)
de Mooij	Median (range)		RD: 5.9 (1.2–17.6) pCR: 5.3 (1.5–18.7)	RD: 6.8 ± 4.1 pCR: 6.6 ± 4.4	48.1% (25/52)
Tatar	Mean ± SD	8.1 ± 4.3	7.2 ± 3.6	7.2 ± 3.6	47.7% (21/44)

ALNs, axillary lymph nodes; pCR, pathologic complete response; RD, residual disease; SUVmax, maximum standardized uptake value.

The mean baseline axillary SUVmax (±SD) ranged from 6.0 (±5.6) to 7.3 (±6.2) [11–13,17] (Table 2). Differentiated per clinical subtype, the mean baseline axillary SUVmax (±SD) ranged in the HER2+ subtype from 5.4 (±3.3) to 11.0 (±3.5) [13,15–17], in the TN subtype from 9.0 (±5.4) to 9.7 (±2.8) [13,15,17], and in the ER+/HER2− subtype from 3.4 (±1.3) to 6.4 (±3.4) [13,17] (Table 3).

**Table 3:** Maximum standardized uptake values of axillary lymph nodes divided by clinical subtype with corresponding axillary pathologic complete response rates

Author	Data type	Baseline SUVmax axilla per subtype	Baseline SUVmax axilla recalculated to mean ± SD	Axillary pCR (%)
Groheux	Mean ± SD	HER2+: 8.2 ± 5.8	HER2+: 8.2 ± 5.8	
Koolen	Median (range)	HER2+: 5.0 (2.2–21) ER+/HER2−: 5.7 (1.8–16) TN: 7.5 (2.5–23)	HER2+: 6.8 ± 4.8 ER+/HER2−: 6.4 ± 3.4 TN: 9.0 ± 5.4	HER2+: 83% (20/24) ER+/HER2−: 3% (1/34) TN: 41% (9/22)
de Mooij	Median (range)	HER2+/TN: RD: 7.5 (1.7–17.6) pCR: 4.5 (2.4–18.7) ER+/HER2−: RD: 4.7 (1.2–10.2) pCR: 5.4 (1.5–11.3)	HER2+/TN: RD: 8.0 ± 4.0 pCR: 6.1 ± 4.2 ER+/HER2−: RD: 5.0 ± 2.3 pCR: 5.7 ± 2.5	HER2+: 83.3% (10/12) TN: 46.7% (7/15) ER+/HER2−: 32% (8/25)
van Ramshorst	Median (IQR)	TN: 8.0 (4.9–13.8) HER2+: 5.3 (3.3–7.6)	TN: 9.0 ± 6.8 HER2+: 5.4 ± 3.3	TN: 47% (21/45) HER2+: 75% (45/60)
Tatar	Mean ± SD	HER2+: 11.0 ± 3.5 TN: 9.7 ± 2.8 Lum A: 3.4 ± 1.3 Lum B: 6.7 ± 2.6	HER2+: 11.0 ± 3.5 TN: 9.7 ± 2.8 Lum A: 3.4 ± 1.3 Lum B: 6.7 ± 2.6	

ER, estrogen receptor; HER2, human epidermal growth factor receptor 2; IQR, interquartile range; Lum, Luminal; pCR, pathologic complete response; RD, residual disease; SUVmax, maximum standardized uptake value; TN, triple negative.

### Axillary pathologic complete response rate

The overall axillary pCR rate ranged from 38% to 48.1% [11–14,17] (Table 2). Differentiated per clinical subtype, the axillary pCR rates were highest in the HER2+ subtype (75–83.3%) [13–15], followed by the TN subtype (41–47%) [13–15] and ER+/HER2− subtype (3–32%) [13,14] (Table 3).

### Association between baseline primary tumor maximum standardized uptake values and axillary pathologic complete response

No study reported on the association between baseline primary tumor SUVmax and axillary pCR, neither overall nor per clinical subtype.

### Association between baseline axillary maximum standardized uptake values and axillary pathologic complete response

Only de Mooij *et al*. associated baseline axillary SUVmax with axillary pCR overall and per clinical subtype. Overall, they did not find a significant difference in baseline axillary SUVmax [median (range)] between patients with residual disease and axillary pCR [5.9 (1.2–17.6) vs. 5.3 (1.5–18.7); *P* = 0.301]. Differentiated per intrinsic subtype, they showed that for HER2+ and TN tumors, the mean baseline axillary SUVmax [median (range)] was significantly higher in patients with axillary residual disease [7.5 (1.7–17.6)] compared with patients with axillary pCR [4.5 (2.4–18.7); *P* = 0.040]. In patients with ER+/HER2− breast tumors, the mean baseline axillary SUVmax [median (range)] was slightly higher in patients with axillary pCR [5.4 (1.5–11.3)] compared with patients with axillary residual disease [4.7 (1.2–10.2)], although this difference did not reach statistical significance (*P* = 0.887) (Table 3) [14]. Three studies reported AUC values of baseline axillary SUVmax to predict axillary pCR, being 0.52 [95% confidence interval (CI): 0.39–0.65; all subtypes included], 0.73 (95% CI not reported; only HER2+), and 0.74 (95% CI: 0.53–0.95; HER2+ and TN) [13,14,16]. de Mooij *et al*. showed that at a baseline axillary SUVmax cutoff of 7.07, sensitivity, specificity, PPV, and NPV values of 90%, 71%, 64%, and 92% were achieved.

## Discussion

To our knowledge, this is the first systematic review to assess whether the degree of metabolic uptake on baseline [^18^F]FDG PET/CT in the primary tumor and ALNs is associated with axillary pCR in cN+ breast cancer patients treated with NST. Based on the limited number of studies in this review, no association could be reported between the baseline primary tumor SUVmax and axillary pCR, neither overall nor in subgroup analysis per clinical subtype. Regarding baseline axillary SUVmax, we found that studies reporting AUC values (with different PET reconstructions) differentiated by clinical subtype (HER2+ and TN) showed higher AUC values than the study that reported an overall AUC value for all subtypes. This might provide an argument to pay attention to clinical subtypes in the interpretation of ALNs on baseline [^18^F]FDG PET/CT. Due to the limited number of studies in this review, however, external validation is required.

Regarding the baseline SUVmax in the primary tumor, there is limited evidence on the association between the degree of SUVmax and axillary pCR in cN+ patients. Groheux *et al*. [18] found that in TN patients (both cN0 and cN+) with residual disease, the primary tumor SUVmax at baseline ranged from 7.0 to 14.0 compared with a range from 8.5 to 18.6 in TN patients with pCR of the primary tumour and ALNs. Another study showed the mean SUVmax of the primary tumor at baseline in patients with residual disease to be 7.8 and in patients with pCR of the primary tumour and ALNs to be 8.7 [19]. Moreover, a study by Koolen *et al*. [20] showed that a higher baseline SUVmax uptake in the primary tumor was observed in tumors with prognostically unfavorable characteristics. Other studies also confirmed that primary breast tumors with relatively high SUVmax on [^18^F]FDG PET/CT are significantly associated with poorer survival [21,22]. In our review concerning cN+ patients, no studies reported the baseline SUVmax of the primary tumor in association with axillary pCR, which demonstrates the current lack of knowledge of this association.

Regarding the baseline SUVmax in ALNs, a few studies showed that cN+ patients with lower axillary SUVmax values on baseline [^18^F]FDG PET/CT in general achieve axillary pCR more frequently [14,23]. Rousseau *et al*. [23] found a significantly lower median SUVmax in cN+ patients with axillary pCR [1.9 (range: 1.5–13.1)] compared with those without pCR [7.1 (range: 1.9–16.3; *P* < 0.001)]. In the study of de Mooij *et al*. [14], a lower median baseline axillary SUVmax of 5.3 (range: 1.5–18.7) was found in cN+ patients who achieved axillary pCR compared with those with residual disease [5.9 (range: 1.2–17.6)]; however, this difference was not statistically significant (*P* = 0.301).

This review demonstrates that baseline axillary SUVmax varies per clinical subtype, with lower baseline axillary SUVmax in the ER+/HER2− subtype (range: 3.4–6.4) and higher baseline axillary SUVmax in HER2+ (range: 5.4–11) and TN subtypes (range: 9.0–9.7) [13–17,24]. Considering axillary pCR rates, this review showed that ER+/HER2− patients achieve axillary pCR less often compared with HER2+ and TN subtypes, which is in line with previously reported axillary pCR rates [3]. Based on the results of de Mooij *et al*., a lower baseline axillary SUVmax may be associated with a higher probability to achieve axillary pCR; however, this does not seem to apply for the ER+/HER2− subtype whereas it may potentially apply for the HER2+ and TN subtype.

Moreover, AUC values of baseline axillary SUVmax to predict axillary pCR were 0.52 (95% CI: 0.39–0.65), 0.73, and 0.74 (95% CI: 0.53–0.95). In the study reporting a low AUC value of 0.52, patients with ER+/HER2−, TN, and HER2+ subtypes were included. Both studies with higher AUC values (0.73 and 0.74) included only HER2+ and TN patients. This indicates that the ability of baseline axillary SUVmax to predict axillary pCR may enhance when differentiating between clinical subtypes.

This systematic review has some limitations. First, data pooling of SUVmax for both primary tumor and ALNs was not possible due to the limited number of included studies. In addition, most studies did not report adherence to EARL guidelines (standardized recommendations for PET/CT reconstructions, developed by the European Association of Nuclear Medicine). Therefore, as all studies were carried out in different institutions using different PET/CT reconstructions, SUVmax may not be comparable between studies, and only ranges of SUVmax are reported in this review. Moreover, only one of the seven studies associated baseline axillary SUVmax with axillary pCR rates in general, and differentiated per clinical subtype. Second, the included studies vary in the type of axillary surgery procedures and therefore axillary pCR percentages may differ depending on the used procedure. Third, the focus of this review was on PET/CT with [^18^F]FDG as a PET tracer. In recent years, other tumor-specific tracers became available in breast cancer patients, such as fibroblast activating protein inhibitor, fluoroestradiol, and anti-1-amino-3-fluorocyclobutane-1-carboxylic acid, which may hold potential future implications. Due to limited evidence about these new PET tracers in cN+ breast cancer patients at baseline, however, these were not incorporated in this review.

## Conclusion

Based on current evidence, no association between the primary tumor SUVmax on baseline [^18^F]FDG PET/CT and axillary pCR was found. There might be a potential association between baseline axillary SUVmax and the probability to achieve axillary pCR differentiated between clinical subtypes. Future studies should differentiate baseline metabolic uptake in primary tumor and ALNs between patients with axillary pCR and axillary residual disease after NST differentiated per clinical subtype, to potentially improve the baseline [^18^F]FDG PET/CT reporting and to determine the probability of NST response.

## Acknowledgements

The authors wish to thank G.H.L.M. Franssen for his assistance with the literature search used to conduct this systematic review.

### Conflicts of interest

M.L. received a salary from Kankeronderzoeksfonds Limburg. F.J.G.A. received a salary from the Dutch Cancer Society (REFINE-trial; project 14055). S.M.E.G. and V.C.G.T.H. report institutional grants from Roche, Pfizer, Novartis, Eli Lilly, Daiichi Sankyo, AstraZeneca, and Gilead outside the submitted work. M.L.S. received institutional research funding not related to this study from Servier, Pharma, Nutricia, and Illumina for the microbiota study. T.J.A.N. received speaker honoraria and institutional grant support from Bayer and GE Healthcare, not related to the content of this study. For F.M.M., there are no conflicts of interest.

## Supplementary Material


