# Supplementary material for: A review of the association between baseline [18F] fluorodeoxyglucose uptake and axillary pathological complete response in node-positive breast cancer patients: focus on clinical subtypes
Source: Nucl Med Commun. 2025 Feb 24;46(5):384–91. doi: 10.1097/MNM.0000000000001964 (PMC11964195; doi:10.1097/MNM.0000000000001964)
Supplement: Supplementary file 1 [file nmc-46-384-s001.pdf]

Table S1. Pubmed search up to July 31st, 2024

#1	"Breast Neoplasms"[MeSH] OR (Breast[MeSH] OR mamma*[tiab] OR breast*[tiab]) AND (Neoplasms[MeSH] OR "Neoplasm Staging"[MeSH] OR Tumour*[tiab] OR carcinoma*[tiab] OR neoplas*[tiab] OR tumor*[tiab] OR oncolog*[tiab] OR malignan*[tiab] OR cancer*[tiab])	Results # 585,528
#2	"18F-FDG PET/CT"[tiab] OR "18 F-FDG PET/CT"[tiab] OR "(18)F-FDG-PET"[tiab] OR "FDG-PET/CT"[tiab] OR (("Positron Emission Tomography Computed Tomography"[MeSH] OR PET/CT[tiab] OR "positron emission tomograph*"[tiab]) AND ("Fluorodeoxyglucose F18"[MeSH] OR FDG[tiab] OR "Fluorodeoxyglucose F18"[tiab] OR "2-Fluoro-2-deoxy-D-glucose"[tiab] OR "2 Fluoro 2 deoxy D glucose"[tiab] OR "2-Fluoro-2-deoxyglucose"[tiab] OR "Fluorodeoxyglucose 18F"[tiab] OR "18F Fluorodeoxyglucose"[tiab] OR "Fluorine-18-fluorodeoxyglucose"[tiab] OR "Fludeoxyglucose F 18"[tiab] OR Fluorodeoxyglucose[tiab] OR fludeoxyglucose[tiab]) AND ("standard uptake value*"[tiab] OR SUV[tiab] OR "standardized uptake value*"[tiab]))	Results # 1873
#3	"axillary lymph node metastasis"[tiab] OR "axillary lymph nodes metastases"[tiab] OR ((Axilla[MeSH] OR axilla*[tiab]) AND ("Lymph Nodes"[MeSH] OR "Lymphatic Metastasis"[MeSH] OR "node-positive*"[tiab] OR "lymph node*"[tiab] OR "node lymph"[tiab] OR "nodal stag*"[tiab]))	Results # 323

Table S2. Embase Search up to July 31st, 2024

1	exp "breast tumor"/	
2	exp breast/	
3	mamma*.ti,ab,kw.	
4	breast*.ti,ab,kw.	
5	2 or 3 or 4	1302029
6	exp neoplasm/	
7	exp "carcinoma"/	
8	exp "oncology"/	
9	exp "cancer staging"/	
10	tumour*.ti,ab,kw.	
11	carcinoma*.ti,ab,kw.	
12	neoplas*.ti,ab,kw.	
13	tumor*.ti,ab,kw.	
14	oncolog*.ti,ab,kw.	
15	malignan*.ti,ab,kw.	
16	cancer*.ti,ab,kw.	
17	6 or 7 or 8 or 9 or 10 or 11 or 12 or 13 or 14 or 15 or 16	7149933
18	5 and 17	769064
19	1 or 18	883553
20	"18F-FDG PET-CT".ti,ab,kw.	
21	"18 F-FDG PET-CT".ti,ab,kw.	
22	"FDG-PET-CT".ti,ab,kw.	
23	20 or 21 or 22	35082
24	exp "positron emission tomography-computed tomography"/	
25	PET-CT.ti,ab,kw.	
26	positron emission tomograph*.ti,ab,kw.	
27	24 or 25 or 26	192181
28	exp "fluorodeoxyglucose f 18"/	
29	FDG.ti,ab,kw.	
30	"fluorodeoxyglucose F18".ti,ab,kw.	
31	"2-Fluoro-2-deoxy-D-glucose".ti,ab,kw.	
32	"2-Fluoro-2-deoxyglucose".ti,ab,kw.	
33	"fluorodeoxyglucose 18F".ti,ab,kw.	
34	"18F Fluorodeoxyglucose".ti,ab,kw.	
35	"Fluorine-18-fluorodeoxyglucose".ti,ab,kw.	
36	"Fludeoxyglucose F 18".ti,ab,kw.	

37	Fluorodeoxyglucose.ti,ab,kw.	
38	Fludeoxyglucose.ti,ab,kw.	
39	28 or 29 or 30 or 31 or 32 or 33 or 34 or 35 or 36 or 37 or 38	119302
40	exp "standardized uptake value"/	
41	"standard uptake value".ti,ab,kw.	
42	SUV.ti,ab,kw.	
43	"standardized uptake value".ti,ab,kw.	
44	40 or 41 or 42 or 43	41357
45	27 and 39 and 44	20635
46	23 or 45	43514
47	"axillary lymph node metastasis".ti,ab,kw.	
48	"axillary lymph nodes metastases".ti,ab,kw.	
49	47 or 48	1943
50	exp axilla/	
51	axilla.ti,ab,kw.	
52	50 or 51	19961
53	exp "lymph node"/	
54	exp "lymph node metastasis"/	
55	"node-positive".ti,ab,kw.	
56	"lymph node".ti,ab,kw.	
57	"node lymph".ti,ab,kw.	
58	"nodal stag*".ti,ab,kw.	
59	53 or 54 or 55 or 56 or 57 or 58	443460
60	52 and 59	7658
61	49 or 60	9311
62	19 and 46 and 61	213
